# Comprehensive analysis of a stochastic wireless sensor network motivated by Black-Karasinski process

**DOI:** 10.1038/s41598-024-59203-3

**Published:** 2024-04-16

**Authors:** Peijiang Liu, Anwarud Din

**Affiliations:** 1 School of Statistics and Mathematics, Guangdong University of Finance and Economics, Guangzhou, 510320 People’s Republic of China; 2 Department of Mathematics, Sun Yat-sen University, Guangzhou, 510275 People’s Republic of China

**Keywords:** Sensor networks, Epidemic model, Wireless sensor networks, Noise, Control technique, Stationary distribution, Mathematics and computing, Physics

## Abstract

Wireless sensor networks (WSNs) encounter a significant challenge in ensuring network security due to their operational constraints. This challenge stems from the potential infiltration of malware into WSNs, where a single infected node can rapidly propagate worms to neighboring nodes. To address this issue, this research introduces a stochastic $\textsf{S}\textsf{E}\textsf{I}\textsf{R}\textsf{S}$ model to characterize worm spread in WSNs. Initially, we established that our model possesses a globally positive solution. Subsequently, we determine a threshold value for our stochastic system and derive a set of sufficient conditions that dictate the persistence or extinction of worm spread in WSNs based on the mean behavior. Our study reveals that environmental randomness can impede the spread of malware in WSNs. Moreover, by utilizing various parameter sets, we obtain approximate solutions that showcase these precise findings and validate the effectiveness of the proposed $\textsf{S}\textsf{E}\textsf{I}\textsf{R}\textsf{S}$ model, which surpasses existing models in mitigating worm transmission in WSNs.

## Introduction

The progress of information technology has brought about an increase in alarming incidents related to wireless networks^1,2^. This progress in the field not only created security issues and threats to the entire globe but also endangered human beings^3^. Within a wireless network, a sensor node is a small, intelligent, and cost-effective device^4^. WSNs are utilized for collecting periodic data in various deployments, including mission-critical scenarios, these networks have numerous significant applications, such as object monitoring in agriculture, military target tracking, disaster management, environmental and pollution monitoring, exploration of dangerous environments, flood detection, traffic monitoring, vehicle tracking, gas monitoring, seismic sensing, water quality monitoring, and healthcare applications (Akyildiz, Su, Sankarasubramaniam^5–7^. Nevertheless, sensor nodes are not only low-cost devices but also operate intelligently. However, they are subject to resource constraints, such as limited battery life, memory capacity, and processing capabilities^8,9^. Hence, the limited resources and decentralized architecture of wireless sensor networks make it highly challenging to establish wireless communication and ensure adequate security provisions between these networks. Wireless networks are more susceptible to threats compared to other networks, as they exhibit greater vulnerability^10^. Despite implementing various security mechanisms to protect the network, software glitches and vulnerabilities are common challenges that can be exploited by hackers. Software vulnerabilities arise from various sources, including coding errors, design flaws, or insufficient security measures implemented in software applications or systems. These concerns become even more critical in the context of WSNs. Sensor nodes in WSNs have limited communication range and rely on multi-hop data delivery^11^. As a result of these limitations, nodes in the networks have limited defense capabilities against virus attacks, including malicious signals, worms, viruses, and more^12^. Controlling the propagation of worms is crucial for the network’s sustainability. Therefore, the study of malicious signal transmission and mathematical modeling plays a crucial role in understanding and mitigating these threats^13–16^.

Mathematical modeling techniques have been widely recognized as the most essential and straightforward tools for studying and predicting the dynamics of various epidemic diseases^17–21^. It is observed that various random processes particularly related to the environment like humidity, rainfall, temperature, and many other factors have a significant impact on the dynamic behavior of different epidemic diseases. These effects allow researchers to include random processes in the traditional ODE models which helps to elucidate the impact of environmental fluctuations. This variability can arise from variations in values of the parameters or the introduction of stochastic noises into the underlying systems^22–24^. Additionally, random models offer an additional level of freedom and are very close to reality compared to their ODE counterparts. Numerous authors have extensively investigated the dynamics of various populations using perturbations in the form of Brownian motion or white noises, for instance, one can see^25,26^ and references cited therein.

In a real-world scenario, the worms spreading across a wireless sensor network could be illustrated through a hypothetical situation involving a network of interconnected sensors deployed for monitoring and collecting data in a critical infrastructure setting, such as a smart city, industrial plant, or environmental monitoring system. Here’s a fictional example to help illustrate the scenario. As the worm spreads, it starts causing disruptions within the sensor network. It may manipulate sensor readings, leading to inaccurate data being sent to the central control system. This can result in misinformed decisions and potentially disrupt industrial processes^27,28^.

This article represents a significant advancement in effectively demonstrating the utility of the $\textsf{S}\textsf{E}\textsf{I}\textsf{R}\textsf{S}$ model, which can assist researchers in accurately elucidating the dynamics of malware propagation within WSNs. By incorporating white noise perturbations, we aim to uncover the impacts of environmental variations and parameter variability on the propagation process. The study elucidates the interplay of metamorphism among the network nodes, shedding light on the relationship. By leveraging the concepts of stochastic epidemic theory, the $\textsf{S}\textsf{E}\textsf{I}\textsf{R}\textsf{S}$ model is conceptualized and applied to investigate the dynamics of malware. The suggested model is validated and rigorously demonstrated via numerous simulations, providing explicit verification of its effectiveness.

The remaining parts of the manuscript are organized as below. In Sect. "Proposed model", we extend the model to the stochastic epidemic model on the transmission of worms in wireless sensor networks. In Sect. "Qualitative analysis of positive solution", the dynamical features of the globalized positive model’ solution are given. In Sect. "Extinction analysis of the worm-free equilibrium", we demonstrate that the worm disease exhibits exponential extinction under specific conditions. In Sect. "Ergodic stationary distribution", we establish the sufficient conditions necessary for the existence of an ergodic stationary distribution. The theory of the obtained results is qualitatively and quantitatively verified, and given their numerical simulation in Sect. "Numerical simulations". The analysis is completed in Sect. "Conclusion" with the concluding remarks and further research directions are suggested.

## Proposed model

**Figure 1:**
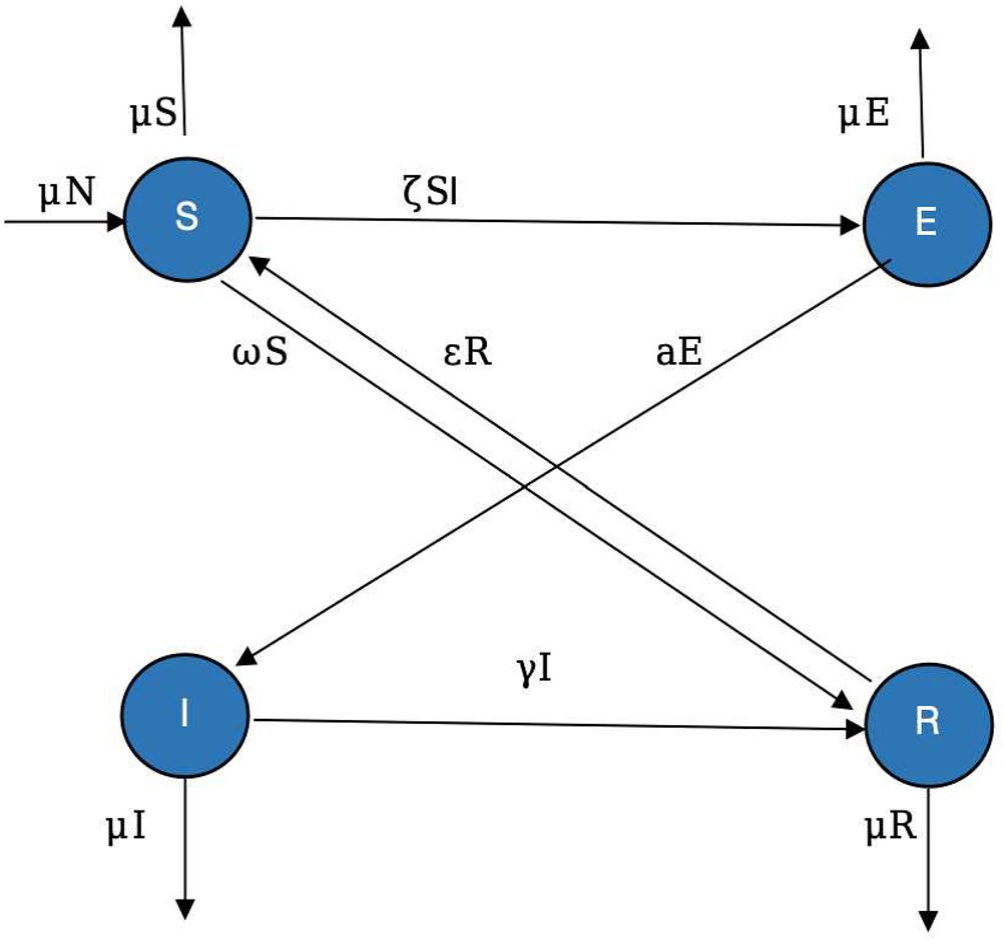
The chart shows the flow of nodes in various states of the system (1).

Recently Ojha et al.^29^ constructed a problem using the approach of a deterministic version of the wireless sensor networks epidemic model. Worms spreading across a wireless sensor network could be illustrated through a hypothetical situation involving a network of interconnected sensors deployed for monitoring and collecting data in a critical infrastructure setting, such as a smart city, industrial plant, or environmental monitoring system. A network is formulated consisting of ${{\textsf {N}}}$ nodes at any time *t* and these nodes are distributed uniformly across the specified area. All of these nodes are uniformly scattered in the area $L^2$ with average density ($\rho$) and *r* and hence the covering region for sensing is $\pi r^2$. To establish communication, it is required that one node should exist in this covering area. Once a node gathers some information, the same must be circulated in the neighboring nodes or it should be directly sent to the sink. It will be assumed that all of the network nodes are vulnerable to the virus assaults and can catch the worm. The dynamics of the nodes from one stage into another based on the worm is shown in Fig. 1. The second main assumption is that all of the nodes can mix homogeneously, that is, information may be circulated by a node to any other node that lies within the sensing area.

To formulate the model, we shall divide the entire nodes of the network into four compartments based on their worm status. These classes are:

1. The vulnerable nodes $\textsf{S}(t)$ which are exposed to the worm assaults and are currently safe.
2. The latent/exposed nodes $\textsf{E}(t)$, nodes which encountered by the worms but not infectious.
3. The infectious nodes $\textsf{I}(t)$ the nodes that are infected by the worm and can spread the worm to other nodes.
4. The recovered nodes $\textsf{R}(t)$, that have been equipped with a detection tool capable of identifying and removing worm infection. nodes.

Every node will be able to spread the malware within the region $\pi r^2$ for a given sensing area of radius *r*. The notion $\rho (t)=\frac{S(t)}{L \times L}$ stands for the density of per unit areal susceptibility of the nodes inside the networks. The size of nodes in the vicinity of the sensing node is designated and defined as $S^{\prime }(t)=\frac{S(t) \pi r^2}{L^2}$. For convenience, let $\zeta =\frac{\pi r^2}{L^2} \beta$ represent a parameter. The model is as follows:

1 $$\begin{aligned} \begin{aligned} \frac{d \textsf{S}(t)}{d t}&=-\mu {{\textsf {N}}}\zeta \textsf{S}(t) \textsf{I}(t)+\varepsilon \textsf{R}(t)-(\mu +\omega ) \textsf{S}(t), \\ \frac{d \textsf{E}(t)}{d t}&=\zeta \textsf{I}(t)\textsf{S}(t)-(a+\mu )\textsf{E}(t), \\ \frac{d \textsf{I}(t)}{d t}&=a \textsf{E}(t)-(\gamma +\mu ) \textsf{I}(t), \\ \frac{d \textsf{R}(t)}{d t}&=\gamma \textsf{I}(t)+\omega \textsf{S}(t)-(\varepsilon +\mu ) \textsf{R}(t). \end{aligned} \end{aligned}$$

A detailed interpretation of the model parameters is presented in Table 1.

**Table 1:** Parameters of the model and their interpretation.

Symbol	Symbols description
$\varepsilon$	Denotes the re-vulnerability of the removed nodes
$\mu$	Represent the rate at which nodes lose their energy and hence assumed dead
$\beta$	The rate of transmitting the worm-present information by infected nodes to susceptible nodes which develop infection therein
*a*	Stand for the rate at which exposed nodes go to the infected class
$\gamma$	The rate of removal/recovery
$\omega$	The rate at which vulnerable nodes get recovery

By using the techniques of differential equations, one can obtain the worm-free equilibrium (WFE):

2 $$\begin{aligned} \P _0=\left( \textsf{S}^0, \textsf{E}^0, \textsf{I}^0, \textsf{R}^0\right) =\left( \frac{(\varepsilon +\mu ) {{\textsf {N}}}}{(\varepsilon +\mu +\omega )}, 0,0, \frac{ \mu {{\textsf {N}}}}{(\varepsilon +\mu +\omega )}\right) . \end{aligned}$$

Following the well-known procedure for calculating the threshold quantity for an ODE system, one can obtain the value of ${\mathbb {R}}_0$ as

$$\begin{aligned} {\mathbb {R}}_0=\frac{{{\textsf {N}}}\zeta (\varepsilon +\mu )}{(a+\mu )(\gamma +\mu )(+\omega +\varepsilon +\mu )}. \end{aligned}$$

Status of the phenomenon where worms are present in the WSN is called the endemic equilibrium (EE) or the worm-present equilibrium and is described as $\P ^*=\left( \textsf{S}^*, \textsf{E}^*, \textsf{I}^*, \textsf{R}^*\right)$ where

3 $$\begin{aligned} \begin{aligned} \textsf{S}^*&=\frac{1}{ {\mathbb {R}}_0}\frac{{{\textsf {N}}}(\varepsilon +\mu ) }{(\varepsilon +\omega +\mu )}, \\ \textsf{E}^*&=\left( 1-\frac{1}{ {\mathbb {R}}_0}\right) \frac{\zeta {{\textsf {N}}}(\varepsilon +\mu )(\gamma +\mu )}{\{(\omega +\mu +\varepsilon )(\mu +\alpha )+ \varepsilon \mu \gamma \}}, \\ \textsf{I}^*&=\left( 1-\frac{1}{ {\mathbb {R}}_0}\right) \frac{ {{\textsf {N}}}\zeta (+\mu +\varepsilon )}{(+\mu +\alpha )(\mu +\omega +\varepsilon )+ \varepsilon \mu \gamma },\\ \textsf{R}^*&=\frac{ S^* \omega +\gamma \psi ^*}{\varepsilon +\mu }. \end{aligned} \end{aligned}$$

System (1) has always the worm-free equilibrium point given by (2) and has a unique endemic equilibrium (3) whenever ${\mathbb {R}}_0>1$.

Next, we have to prove the local analysis of these equilibria, that is, we will check the behavior of the solution in the long run when the initial data is sufficiently close to the equilibrium points. First of all, we will show the local analysis of the WFE.

### Theorem 1

The worm-free equilibrium point given by (2) is locally asymptotically stable for ${\mathbb {R}}_0<1$ and unstable otherwise.

### Proof

The variational matrix at the WFE is given by

4 $$\begin{aligned} J(\P _0)=\left( \begin{array}{c c c c} -(\omega +\mu )&{}0&{}-\zeta \textsf{S}^0&{}\varepsilon \\ 0&{}-(\mu +a)&{}\zeta \textsf{S}^0&{}0\\ 0&{}a&{}-(\gamma +\mu )&{}0\\ \omega &{}0&{}\gamma &{}-(\varepsilon +\mu ) \end{array} \right) , \end{aligned}$$

The variational matrix (4) has the eigenvalues: $t_1=-(\mu +\omega )$, $t_2=-(\mu +\varepsilon )$ where the remaining two eigenvalues are the roots of the quadratic equation

5 $$\begin{aligned} t^2+(2\mu +\gamma +a)t+(\mu +\gamma )(\mu +a)(1- {\mathbb {R}}_0)=0. \end{aligned}$$

If ${\mathbb {R}}_0<1$, then by Descartes’s rule of sign, relation (5) has no real positive or complex solution with positive real parts. Further, the rule ensures that both the roots are less than zero or complex with negative real parts. Thus, all of the eigenvalues of the variational matrix are complex with negative real parts or negative and thus the WFE is LAS under ${\mathbb {R}}_0<1$. $\square$

Next, we will present the local asymptotic stability of the worm present a fixed point.

### Theorem 2

The worm endemic equilibrium point given by (3) is locally asymptotically stable for ${\mathbb {R}}_0>1$ and otherwise unstable.

### Proof

The variational matrix at the worm’s present equilibrium is given by

6 $$\begin{aligned} J(\P ^*)=\left( \begin{array}{c c c c} -\zeta \textsf{I}^*-(\omega +\mu )&{}0&{}-\zeta \textsf{S}^*&{}\varepsilon \\ \zeta \textsf{I}^*&{}-(\mu +a)&{}\zeta \textsf{S}^*&{}0\\ 0&{}a&{}-(\gamma +\mu )&{}0\\ \omega &{}0&{}\gamma &{}-(\varepsilon +\mu ) \end{array} \right) , \end{aligned}$$

The characteristic equation of matrix (6) is given by

7 $$\begin{aligned} t^4+\chi _1t^3+\chi _2t^2+\chi _3t+\chi _4=0, \end{aligned}$$

where

$$\begin{aligned} \chi _1= & {} \frac{(4\mu +\varepsilon +a +\omega + \gamma ){\mathcal {Q}}_1 {\mathbb {R}}_0+aN\zeta ^2 (\varepsilon +\mu )( {\mathbb {R}}_0-1)}{{\mathcal {Q}}_1 {\mathbb {R}}_0},\\ \chi _2= & {} \frac{{\mathcal {Q}}_1(\varepsilon +\mu )a\zeta N+aN\zeta ^2 (\varepsilon +\mu )(\varepsilon +\omega + \mu )(3\mu +\varepsilon +a+\gamma +\varepsilon )( {\mathbb {R}}_0-1)(\varepsilon +\omega + \mu ) {\mathbb {R}}_0{\mathcal {Q}}_1{\mathcal {Q}}_2}{{\mathcal {Q}}_1 {\mathbb {R}}_0},\\ \chi _3= & {} \frac{{\mathcal {Q}}_1{\mathcal {Q}}_3 {\mathbb {R}}_0+{\mathcal {Q}}_1a\zeta N(\varepsilon +\mu )(\varepsilon +2\mu +\omega )+{\mathcal {Q}}_4a\zeta ^2N (\varepsilon +\mu )(\varepsilon +\omega + \mu )( {\mathbb {R}}_0-1)}{{\mathcal {Q}}_1 {\mathbb {R}}_0},\\ \chi _4= & {} \frac{{\mathcal {Q}}_5{\mathcal {Q}}_1 {\mathbb {R}}_0(\varepsilon +\omega + \mu )+{\mathcal {Q}}_6a\zeta N (\varepsilon +\mu )+{\mathcal {Q}}_1a\zeta ^2N (\varepsilon +\omega + \mu )\mu ( {\mathbb {R}}_0-1)}{{\mathcal {Q}}_1 {\mathbb {R}}_0}, \end{aligned}$$

and

$$\begin{aligned} {\mathcal {Q}}_1= & {} (\varepsilon \mu \omega +\varepsilon +\omega + \mu )(a+\mu ),\\ {\mathcal {Q}}_2= & {} (a+\mu )(\mu +\gamma )+(\varepsilon +\mu )(\mu +\omega )+(2\mu +\gamma +a)(a+\mu )(\mu +\gamma ),\\ {\mathcal {Q}}_3= & {} (2\mu +\gamma +a)\bigg (\varepsilon \omega +(\varepsilon +\mu )(\mu +\omega )\bigg )+(\varepsilon +2\mu +\omega )(a+\mu )(\mu +\gamma ),\\ {\mathcal {Q}}_4= & {} {\mathcal {Q}}_5=(a+\mu )(\mu +\gamma )+(\varepsilon +\mu )(a+2\mu +\gamma ),\\ {\mathcal {Q}}_6= & {} (\mu ^2+\mu \omega +\omega \varepsilon ){\mathcal {Q}}_1. \end{aligned}$$

For ${\mathbb {R}}_0>1$, all of the coefficients $\chi _i$ for $i=1,2,3,4$ of (7) are positive. Further, by calculating ${\mathcal {G}}_1=\chi _1\chi _2-\chi _3>0$ and ${\mathcal {G}}_2=\chi _2\chi _3-\chi _4\chi _1^2>0$ under the same condition of ${\mathbb {R}}_0>1$. Hence, by using the Routh Hurwitz criterion, all of the roots of (7) are negative or complex with negative real parts and hence the worm’s present fixed point is LAS. $\square$

While dealing with real-world scenarios, outbreaks (whether infectious diseases or others) are subject to complex and random variations. Utilizing stochastic models for modeling epidemics may be a more suitable approach, considering the inherent unpredictability of such scenarios. The key purpose of the current work is to introduce the white noise of the model (1). Then the deterministic system (1) may written in the stochastically perturbed format as follows:

8 $$\begin{aligned} \begin{aligned} d \textsf{S}&=\bigg [\mu {{\textsf {N}}}-\zeta \textsf{S}(t) \textsf{I}(t)+\varepsilon \textsf{R}(t)-(\omega +\mu ) \textsf{S}(t)\bigg ]dt+\alpha _1 \textsf{S}(t) d\textsf{Z}_1(t),\\ d \textsf{E}&=\bigg [\zeta \textsf{I}(t) \textsf{S}(t) -(a+\mu )\textsf{E}(t)\bigg ]dt+ \alpha _2\textsf{E}(t) d\textsf{Z}_2(t),\\ d \textsf{I}&=\bigg [a \textsf{E}(t)-(\mu +\gamma ) \textsf{I}(t)\bigg ]dt+\alpha _3 \textsf{I}(t) d\textsf{Z}_3(t), \\ d \textsf{R}&=\bigg [\gamma \textsf{I}(t)+\omega \textsf{S}(t)-(\varepsilon +\mu ) \textsf{R}(t)\bigg ]dt+\alpha _4 \textsf{R}(t) d \textsf{Z}_4(t). \\ \end{aligned} \end{aligned}$$

**Figure 2:**
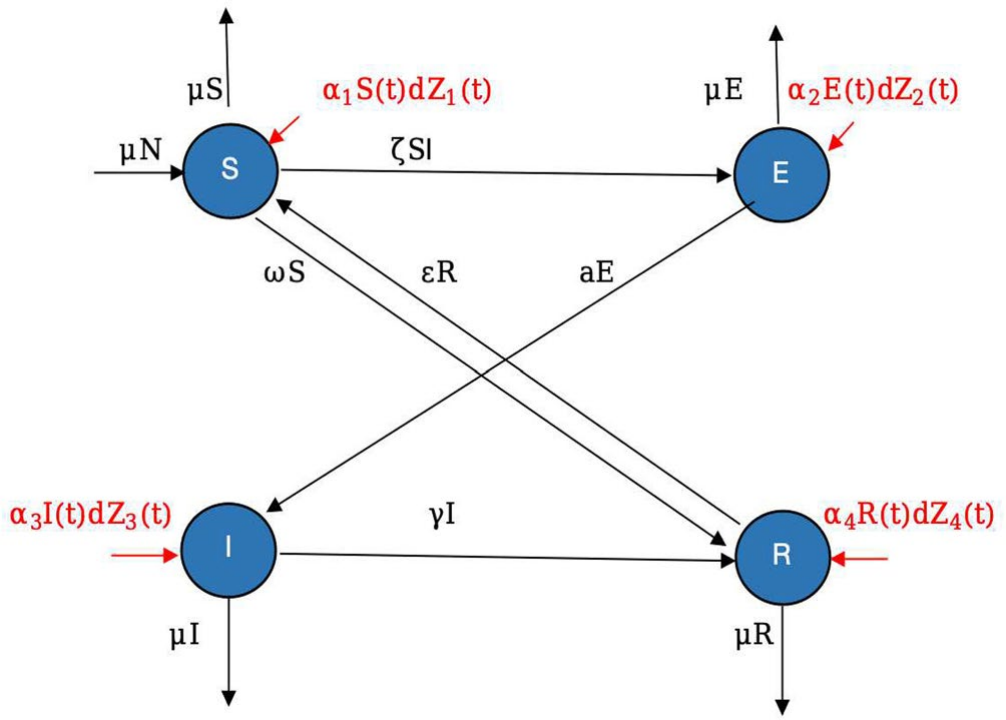
The chart shows the flow of nodes in various states of the system (8).

Where $\textsf{Z}_{i}(t)$ for $i=1,\cdots ,4$ are for the fluctuating dynamics and $\alpha _{1}, \alpha _{2}, \alpha _{3}$ and $\alpha _{4}$ are for the noise intensities. definitely it contains the outcomes of $\textsf{Z}_i(0)=0$ for $i=1,2,\cdots ,4$. Figure 2 represents the diagram of a system (8).

## Qualitative analysis of positive solution

To investigate the dynamic behavior of the system (8), we aim to address the positivity aspects concerning the solution of the proposed system (8) and to prove that the solution of the considered model is unique. The investigation of positive and non-local solutions requires further analysis using Lyapunov function techniques^30,31^.

### Theorem 3

Subject to a non-negative initial state of the variabels, almost surely the global solution $(\textsf{S},\textsf{E},\textsf{I},\textsf{R})(t)\in {\mathbb {R}}^{4}_{+}$ for model (8) exist whenever $t\ge 0$.

### Proof

The coefficient(s) involved in the model (8) are continuous and Lipschitz locally, considering the initial values $(\textsf{S}_0, \textsf{E}_0, \textsf{I}_0, \textsf{R}_0)$ from the space ${\mathbb {R}}_{+}^4$. Consequently, one and only one solution $(\textsf{S}(t), \textsf{E}(t), \textsf{I}(t), \textsf{R}(t))$ (in local sense) exists for *t* belong to the interval $[0, \tau _e)$, here $\tau _e$ represents the explosion-time. To establish the solution behaves like the global, it is necessary to demonstrate that $\tau _e = \infty$ almost surely. Choose a very large number ${\mathcal {K}}_0 \ge 0$ in such a way that $(\textsf{S}(0), \textsf{E}(0), \textsf{I}(0), \textsf{R}(0))$ lies in the strip $\left[ \frac{1}{{\mathcal {K}}_0}, {\mathcal {K}}_0\right]$. For every integer ${\mathcal {K}}_0 \le {\mathcal {K}}$, the stopping-time is defined by the following expression:

$$\begin{aligned} \tau _{\mathcal {K}}=\inf \left\{ t \in \left[ 0, \tau _e\right) : \min (\textsf{S}, \textsf{E}, \textsf{I}, \textsf{R})(t) \le {\mathcal {K}}^{-1} \text{ or } {\mathcal {K}} \le \max \{(\textsf{S}, \textsf{E}, \textsf{I}, \textsf{R})(t)\} \right\} . \end{aligned}$$

Let us define $\infty =\inf \emptyset$, one can notice that $\tau _{e} \ge \tau ^{+}$, which suggests that $\tau ^{+}=\infty$ almost surely, demonstrating that $\tau _{e}=+\infty$ a.s. Assume that $\tau ^{+}$ is $\infty$, then there must be a $T>0$ (real) in such a way that $0<{\mathbb {P}}\left( T>\tau ^{+}\right)$ and

$$\begin{aligned} P\left\{ \tau _{\infty } \le T\right\} >\epsilon . \end{aligned}$$

As a result, there exists a real number ${\mathcal {K}}_0\le {\mathcal {K}}_1$ for which

9 $$\begin{aligned} P\left\{ T\ge \tau _k\right\} >\epsilon ~~\forall ~~ {\mathcal {K}} \ge {\mathcal {K}}_1 \text{. } \end{aligned}$$

To proceed further, let us introduce a function (a $C^2$-function) $V: {\mathbb {R}}_{+}^4 \rightarrow \overline{{\mathbb {R}}}_{+}$

where $$\{x: x \text { is non-negative real number}\}=\overline{{\mathbb {R}}}_{+}$$, by

$$\begin{aligned} \begin{aligned} \textsf{V}(\textsf{S}, \textsf{E}, \textsf{I}, \textsf{R})&= (-1+S-\ln \textsf{S})+(-1+\textsf{E}-\ln \textsf{E})+(-1+\textsf{I}-\ln \textsf{I})+(-1+\textsf{R}-\ln \textsf{R})\\&+\int _{0}^{t}\zeta \textsf{I}(s)ds. \end{aligned} \end{aligned}$$

By employing the Itô formula, we derive

$$\begin{aligned} d\textsf{V}=L\textsf{V}dt+(\textsf{S}-1)\alpha _1d\textsf{Z}_1(t)+(-1+\textsf{E})\alpha _2d\textsf{Z}_2(t)+(-1+\textsf{I})\alpha _3d\textsf{Z}_3(t)+(-1+\textsf{R})\alpha _4d\textsf{Z}_4(t), \end{aligned}$$

where

$$\begin{aligned} \begin{aligned} L\textsf{V}&=(1-\frac{1}{\textsf{S}})(-\zeta \textsf{S}\textsf{I}+\mu {{\textsf {N}}}-(\omega +\mu ) \textsf{S}+\varepsilon \textsf{R})+(1-\frac{1}{\textsf{E}})(\zeta \textsf{I}\textsf{S}-(a+\mu )\textsf{E})\\&+(1-\frac{1}{\textsf{I}})(-(\mu +\gamma ) \textsf{I}+a \textsf{E})+(1-\frac{1}{\textsf{R}})(\omega \textsf{S}+\gamma \textsf{I}-(\mu +\varepsilon ) \textsf{R})\\&+\frac{\alpha _1^2+\alpha _2^2+\alpha _3^2+\alpha _4^2}{2}+\zeta \textsf{I}(0)-\zeta I(t),\\ {}&\le -\zeta \textsf{I}\textsf{S}+\mu {{\textsf {N}}}+\varepsilon \textsf{R}-(+\omega +\mu ) \textsf{S}-\frac{\mu {{\textsf {N}}}}{\textsf{S}}+\zeta \textsf{I}-\frac{\varepsilon \textsf{R}}{\textsf{S}}+(\mu +\omega ) \\ {}&+\zeta \textsf{S}\textsf{I}-(a+\mu )\textsf{E}-\frac{\zeta \textsf{S}\textsf{I}}{\textsf{E}}-(+a+\mu )+a \textsf{E}-(+\mu +\gamma ) \textsf{I}-\frac{a \textsf{E}}{\textsf{I}}+(+\mu +\gamma ) \\ {}&+\gamma \textsf{I}+\omega \textsf{S}-(+\mu +\varepsilon ) \textsf{R}-\frac{\gamma \textsf{I}}{\textsf{R}}-\frac{\omega \textsf{S}}{\textsf{R}}+(\varepsilon +\mu ) +\frac{\alpha _1^2+\alpha _2^2+\alpha _3^2+\alpha _4^2}{2}+\zeta \textsf{I}(0)-\zeta I(t)\\&\le \mu {{\textsf {N}}}+4\mu +\omega +a+\gamma +\varepsilon +\zeta \textsf{I}(0)+\frac{\alpha _1^2+\alpha _2^2+\alpha _3^2+\alpha _4^2}{2}.\\ \end{aligned} \end{aligned}$$

We know that $\textsf{I}(0)\ge 0$, then

10 $$\begin{aligned} L\textsf{V}\le \mu {{\textsf {N}}}+4\mu +\omega +a+\gamma +\varepsilon +\zeta \textsf{I}(0)+\frac{\alpha _1^2+\alpha _2^2+\alpha _3^2+\alpha _4^2}{2}:= M. \end{aligned}$$

Since *M* is real positive and not dependent on the state and independent variables, we have

11 $$\begin{aligned} d \textsf{V}(\textsf{S}, \textsf{E},\textsf{I}, \textsf{R}) \le M d t+(\textsf{S}-1)\alpha _1d\textsf{Z}_1(t)+(\textsf{E}-1)\alpha _2d\textsf{Z}_2(t)+(\textsf{I}-1)\alpha _3d\textsf{Z}_3(t)+(\textsf{R}-1)\alpha _4d\textsf{Z}_4(t). \end{aligned}$$

By taking integral of relation (10) within the interval $[0,T \wedge \tau _k]$ and taking expectations, then we can get

12 $$\begin{aligned} \begin{aligned} G\bigg [H((\textsf{S},\textsf{E},\textsf{I},\textsf{R})(\tau _{\mathcal {K}}\wedge T))\bigg ]&\le \textsf{V}(\textsf{S}_0, \textsf{E}_0, \textsf{I}_0, \textsf{R}_0)+G\bigg [\int ^{\tau _k\wedge T}_0{\mathbb {K}}dt\bigg ],\\&\le H(\textsf{S}_0, \textsf{E}_0, \textsf{I}_0, \textsf{R}_0)+MT<\infty . \end{aligned} \end{aligned}$$

Setting $\Omega _{\mathcal {K}}=\{\tau _{\mathcal {K}}\le T\}$ for ${\mathcal {K}}\ge {\mathcal {K}}_1$ and by Eq. (3), $P(\Omega _{\mathcal {K}})\ge \epsilon$. It can be easily judged that for every $\omega$ in $\Omega _{\mathcal {K}}$, there exist one solution at the point $(\tau _{\mathcal {K}},\omega )$ that is equal to $\frac{1}{{\mathcal {K}}}$ or ${\mathcal {K}}$. Consequently, $H((\textsf{S},\textsf{E},\textsf{I},\textsf{R})(\tau _{\mathcal {K}}))\ge \frac{1}{{\mathcal {K}}}-1+\text {log}{\mathcal {K}}$ or ${\mathcal {K}}-1-\text {log}{\mathcal {K}}$. Therefore,

13 $$\begin{aligned} H((\textsf{S},\textsf{E},\textsf{I},\textsf{R})(\tau _{\mathcal {K}}))\ge \bigg (\frac{1}{{\mathcal {K}}}-1+\text {log}{\mathcal {K}}\bigg )\wedge \big ({\mathcal {K}}-1-\text {log}{\mathcal {K}}\big ). \end{aligned}$$

Utilizing relations (12) and (13), we can write

14 $$\begin{aligned} \begin{aligned} H(\textsf{S}_0, \textsf{E}_0, \textsf{I}_0, \textsf{R}_0)+MT&\ge G\bigg [1_{\Omega (\omega )}H\bigg ((\textsf{S},\textsf{E},\textsf{I},\textsf{R})(\tau _{\mathcal {K}})\bigg )\bigg ]\\&\ge \epsilon \bigg [\bigg (\frac{1}{{\mathcal {K}}}-1+\text {log}{\mathcal {K}}\bigg )\wedge ({\mathcal {K}}-1-\text {log}{\mathcal {K}})\bigg ]. \end{aligned} \end{aligned}$$

In the above $1_{\Omega (\omega )}$ stand for represent the $\Omega$-indicator function. By letting ${\mathcal {K}}\rightarrow \infty$, we arrived at $\infty >H\big (\cdot \big )+MT=\infty$ showing that $\tau _\infty =\infty$ a.s. $\square$

## Extinction analysis of the worm-free equilibrium

This part of the manuscript deals with the investigate of the extinction of system (8) and establish a threshold to determine whether the disease will die out or persist. We will introduce two auxiliary lemmas that plays an important role in the proof of the key assertion of the current section.

### Lemma 1

Let for $t\ge 0$, the notion $\textsf{M}=\{\textsf{M}_t\}$ denotes the real-valued function and it signifies the local martingale that vanishes at $t = 0$. Then

$$\begin{aligned} \lim \limits _{t \rightarrow \infty }\left\langle \textsf{M}, \textsf{M}\right\rangle _{t}=\infty ,~~~~a.s.~~~~\Rightarrow ~~\lim \limits _{t \rightarrow \infty }\frac{\textsf{M}_t}{\left\langle \textsf{M}, \textsf{M}\right\rangle _{t}}=0,~~~a.s.,\\ \end{aligned}$$

and also

$$\begin{aligned} \lim \limits _{t \rightarrow \infty }\sup \frac{\left\langle \textsf{M}, \textsf{M}\right\rangle _{t}}{t}<\infty ,~~~a.s.~~~\Rightarrow ~~~\lim \limits _{t \rightarrow \infty }\frac{\textsf{M}_t}{t}=0,~~~a.s.,\\ \end{aligned}$$

where quadratic variants of $\textsf{M}$ is denoted by the notion $\left\langle \textsf{M}, \textsf{M}\right\rangle _{t}$.

### Lemma 2

(^31^) Assume a solution $(\textsf{S}, \textsf{E}, \textsf{I}, \textsf{R})$ of model (8) subject to an initial data $(\textsf{S}_0, \textsf{E}_0, \textsf{I}_0, \textsf{R}_0) \in {\mathbb {R}}_+^4$, then

$$\begin{aligned} \lim \limits _{t\rightarrow \infty }\frac{\textsf{S}(t)}{t}=0,~~~~~\lim \limits _{t\rightarrow \infty }\frac{\textsf{E}(t)}{t}=0. \lim \limits _{t\rightarrow \infty }\frac{\textsf{I}(t)}{t}=0,~~~~~\lim \limits _{\rightarrow \infty }\frac{\textsf{R}(t)}{t}=0,~~~~~a.s. \end{aligned}$$

Moreover, if $\mu > \frac{\alpha _1^2\vee \alpha _2^2\vee \alpha _3^2\vee \alpha _4^2}{2},$ then

$$\begin{aligned} \lim \limits _{t\rightarrow \infty }\frac{\int _0^t \textsf{S}(s)d \textsf{Z}_1(s)}{t}=0,~~~\lim \limits _{t\rightarrow \infty }\frac{\int _0^t \textsf{E}(s)d \textsf{Z}_2(s)}{t}=0, \\ \lim \limits _{t\rightarrow \infty }\frac{\int _0^t \textsf{I}(s)d \textsf{Z}_3(s)}{t}=0,~~~~~ \lim \limits _{t\rightarrow \infty }\frac{\int _0^t \textsf{R}(s)d \textsf{Z}_4(s)}{t}=0,~~~a.s. \end{aligned}$$

Define the parameter as follows:

15 $$\begin{aligned} {\mathbb {R}}_s=\frac{2a{{\textsf {N}}}(a+\mu )^2}{\bigg \{(a+\mu )^2(\mu +\gamma +\frac{\alpha _3^2}{2}) \wedge a^2\frac{\alpha _2^2}{2}\bigg \}}. \end{aligned}$$

### Theorem 4

If the threshold number ${\mathbb {R}}_0^s<1$, as defined by Eq. (15), the exposed and infected worms ($\textsf{E}(t)$ and $\textsf{I}(t)$ functions) in the system (8) will almost surely tend to zero following an exponential function.

### Proof

Regarding model (8), one can notice that

16 $$\begin{aligned} \begin{aligned} d(\textsf{E}+\textsf{S}+\textsf{R}+\textsf{I})&=[\mu {{\textsf {N}}}-\mu (\textsf{E}+\textsf{S}+\textsf{R}+\textsf{I})]dt+\alpha _1\textsf{S}d\textsf{Z}_1+\alpha _2\textsf{E}dZ_2+\alpha _3\textsf{I}d\textsf{Z}_3+\alpha _4\textsf{R}d\textsf{Z}_4. \end{aligned} \end{aligned}$$

Integrating the above expression within the range 0 and *t*, we get

17 $$\begin{aligned} \frac{S+\textsf{E}+\textsf{I}+\textsf{R}}{t} ={{\textsf {N}}}+\psi _1(t), \end{aligned}$$

where

18 $$\begin{aligned} \begin{aligned} \psi _1&=\frac{1}{\mu }\bigg [\frac{1}{t}(\textsf{E}_0+\textsf{S}_0+\textsf{R}_0+\textsf{I}_0)-\frac{1}{t}(\textsf{E}+\textsf{S}+\textsf{R}+\textsf{I}\bigg ]\\&+\frac{\alpha _1\int _0^t\textsf{S}(s)d\textsf{Z}_1}{t}+\frac{\alpha _2\int _0^t\textsf{E}(s)d\textsf{Z}_2}{t}+\frac{\alpha _3\int _0^t\textsf{I}(s)d\textsf{Z}_3}{t}+\frac{\alpha _4\int _0^t\textsf{R}(s)d\textsf{Z}_4}{t}\bigg ]. \end{aligned} \end{aligned}$$

Now using the concept of Lemma 2, we get

19 $$\begin{aligned} \lim \limits _{t\rightarrow \infty }\sup \left\langle \textsf{S}+\textsf{E}+\textsf{I}+\textsf{R}\right\rangle ={{\textsf {N}}}. \end{aligned}$$

Assume that $(\textsf{S}, \textsf{E}, \textsf{I}, \textsf{R}) \in {\mathbb {R}}_+^4$ be a solution of equations (8) with positive initial values $(\textsf{S}_0, \textsf{E}_0, \textsf{I}_0, \textsf{R}_0) \in {\mathbb {R}}_+^4$. Define

20 $$\begin{aligned} \textsf{B}(t)=a\textsf{E}+(\mu +a)\textsf{I}. \end{aligned}$$

Differentiating Eq. (20) following $It\hat{o}$ formula, one can get

21 $$\begin{aligned} \begin{aligned} d ln\textsf{B}(t)&=\bigg \{\frac{1}{\textsf{B}}\times [a\zeta \textsf{S}\textsf{I}-(a+\mu )(\gamma +\mu ) \textsf{I}-\frac{a^2\alpha _2^2\textsf{E}^2+(a+\mu )^2\alpha _3^2 \textsf{I}^2}{\textsf{B}^2}\bigg \}dt +\frac{a \alpha _2\textsf{E}}{\textsf{B}}d\textsf{Z}_2\\ {}&+\frac{(\mu +a) \alpha _3 \textsf{I}}{\textsf{B}}d\textsf{Z}_3 \\ {}&\le \frac{ a\zeta \textsf{S}}{(a+\mu )} -\frac{(a+\mu )^2(\mu +\gamma ) \textsf{I}^2}{\textsf{B}^2}-\frac{1}{(a\textsf{E}+(+a+\mu )\textsf{I})^2}\bigg \{(a+\mu )^2 \frac{\alpha _3^2}{2}\textsf{I}^2+a^2\frac{\alpha _2^2}{2}\textsf{E}^2\bigg \}dt\\&+\frac{a \alpha _2\textsf{E}}{\textsf{B}}d\textsf{Z}_2+\frac{(\mu +a) \alpha _3 \textsf{I}}{\textsf{B}}d\textsf{Z}_3 \\ {}&\le \frac{ a\zeta \textsf{S}}{(\mu +a)} -\frac{(\mu +a)^2(\gamma +\mu +\frac{\alpha _3^2}{2}) \textsf{I}^2+a^2\frac{\alpha _2^2}{2}\textsf{E}^2}{(a\textsf{E}+(\mu +a)\textsf{I})^2}\bigg \}dt+\frac{a \alpha _2\textsf{E}}{\textsf{B}}d\textsf{Z}_2+\frac{(\mu +a) \alpha _3 \textsf{I}}{\textsf{B}}d\textsf{Z}_3 \\&\le \zeta \textsf{S}dt -\frac{(\textsf{E}^2+\textsf{I}^2)}{(a\textsf{E}+(\mu +a)\textsf{I})^2}\bigg \{(\mu +a)^2(\gamma +\mu +\frac{\alpha _3^2}{2}) \wedge a^2\frac{\alpha _2^2}{2}\bigg \}dt\\&+\frac{a \alpha _2\textsf{E}}{\textsf{B}}d\textsf{Z}_2+\frac{(\mu +a) \alpha _3 \textsf{I}}{\textsf{B}}d\textsf{Z}_3. \end{aligned} \end{aligned}$$

Obviously,

$$\begin{aligned} (a\textsf{E}+(\mu +a)\textsf{I})^2\le 2[a^2\textsf{E}+(\mu +a)^2\textsf{I}^2)\le 2(\mu +a)^2(\textsf{E}^2+\textsf{I}^2). \end{aligned}$$

Therefore, we have

22 $$\begin{aligned} \begin{aligned} d ln\textsf{B}(t)&\le \zeta \textsf{S}dt -\frac{(\textsf{E}^2+\textsf{I}^2)}{(a\textsf{E}+(\mu +a)\textsf{I})^2}\bigg \{(\mu +a)^2(\gamma +\mu +\frac{\alpha _3^2}{2}) \wedge a^2\frac{\alpha _2^2}{2}\bigg \}dt\\&+\frac{a \alpha _2\textsf{E}}{\textsf{B}}d\textsf{Z}_2+\frac{(\mu +a) \alpha _3 \textsf{I}}{\textsf{B}}d\textsf{Z}_3. \\ \end{aligned} \end{aligned}$$

Taking integration of the inequality (22) within the range 0 and *t*, employing Lemma 2 and ${\mathbb {R}}_s<1$, we have

23 $$\begin{aligned} \begin{aligned} \lim \limits _{t\rightarrow \infty }\sup \frac{\ln \textsf{B}(t)}{t}&\le \zeta {{\textsf {N}}}dt -\frac{1}{2(a+\mu )^2}\bigg \{(\mu +a)^2(\mu +\gamma +\frac{\alpha _3^2}{2}) \wedge a^2\frac{\alpha _2^2}{2}\bigg \}dt\\&=\frac{\bigg \{(a+\mu )^2(\mu +\gamma +\frac{\alpha _3^2}{2}) \wedge a^2\frac{\alpha _2^2}{2}\bigg \}}{2(\mu +a)^2}({\mathbb {R}}_s-1), ~~ a.s, \end{aligned} \end{aligned}$$

which shows that

24 $$\begin{aligned} \lim \limits _{t\rightarrow \infty } \textsf{E}(t)=\lim \limits _{t\rightarrow \infty } \textsf{I}(t) = 0. \end{aligned}$$

This demonstrates the stochastic asymptotic behavior of the system and hence, the key aim of the theorem holds valid. $\square$

## Ergodic stationary distribution

Ergodic stationary distribution of nodes is rooted in the study of stochastic processes, where nodes within a network undergo random transitions between different states. The ergodic stationary distribution provides insights into the long-term behavior of the network, emphasizing the idea that, over time, the system explores and represents all possible states with sufficient probability. Ergodicity ensures that, given enough time, the network dynamics explore all possible configurations of node states. In the case of the model (8), the system has an endemic steady state. Thus, in this part of the paper, we utilize the techniques proposed by Khasminskii^32^ to explore that the proposed system has a stationary distribution. Let define

25 $$\begin{aligned} d\textsf{X}(t)=\sum _{r=1}^dg_r(t, \textsf{X}(t))d\textsf{B}_r(t). \end{aligned}$$

The diffusion matrix is

$$\begin{aligned} \Pi (x)=(\Upsilon _{ij}(x)),~~~~\Upsilon _{ij}(x)=\sum _{r=1}^dg_r^j(x)g_r^i(x). \end{aligned}$$

### Lemma 3

Let $\textbf{U} \in {\mathbb {R}}^d$ denote an open bounded domain with $\Gamma$ regular boundary. The domain $\textbf{U}$ possesses the following properties:

1. In the neighborhood of $\textbf{U}$ and within its domain, the smallest eigenvalue of the matrix $\mathbf {A(t)}$ remains bounded away from zero.
2. If $\textbf{x}$ belongs to the space ${\mathbb {R}}^{d} \backslash \textbf{U}$, the average time $\tau$ required to cover the path from $\textbf{x}$ to $\textbf{U}$ is not infinite. Moreover, for every compact subset $K \subset {\mathbb {R}}^{n}$, the quantity $\sup _{x \in K} E^{x} \tau$ is finite. Additionally, if $f(\cdot )$ is a function integrable with respect to the measure “$\cdot$”, then

Then the Markov process $\textsf{X}(t)$ admits a unique ergodic stationary distribution $\pi (\cdot )$ and

$$\begin{aligned} {\mathbb {P}}\left\{ \lim _{T \rightarrow \infty } \frac{1}{T} \int _0^T f(X(t)) d t=\int _{{\mathbb {R}}^d} f(x) \pi (d x)\right\} =1, \forall x \in {\mathbb {R}}^d, \end{aligned}$$

where $f(\cdot )$ is an integrable function with respect to the measure $\pi$.

The threshold parameter in the case of the stochastic model is calculated as

26 $$\begin{aligned} {\mathbb {R}}^s=\frac{ {{\textsf {N}}}a}{\bigg (\mu +a+\frac{\alpha _2^2}{2}\bigg )\bigg (\gamma +\mu +\frac{\alpha _3^2}{2}\bigg )}. \end{aligned}$$

### Theorem 5

Assume that ${\mathbb {R}}_s>1$ and $\mu -\frac{\alpha _1^2\vee \alpha _2^2\vee \alpha _3^2\vee \alpha _4^2}{2}>0,$ then for $(\textsf{S}_0, \textsf{E}_0, \textsf{I}_0, \textsf{R}_0) \in {\mathbb {R}}_+^4,$ system (8) has a unique ergodic stationary distribution $\pi$.

### Proof

To establish the conditions (1) and (2) of Lemma 3, we need to validate them. In order to derive condition (1), we consider the diffusion matrix as follows:

$$\begin{aligned} \Upsilon = \begin{pmatrix} \alpha _1^2\textsf{S}^2 &{} 0 &{} 0 &{} 0 \\ 0 &{} \alpha _2^2\textsf{E}^2 &{} 0 &{} 0 \\ 0 &{} 0 &{} \alpha _3^2\textsf{I}^2 &{} 0 \\ 0 &{} 0 &{} 0 &{} \alpha _4^2\textsf{R}^2\\ \end{pmatrix}. \end{aligned}$$

Irrespective of a compact subset of ${\mathbb {R}}_+^4$, it is to be noted that $\Upsilon$ is positive definite matrix, thus confirming condition (1) of Lemma 3.

Next, we derive condition (2). Consider the $C^2$-operator $\textsf{V}:{\mathbb {R}}_+^4\rightarrow {\mathbb {R}}$ given by:

27 $$\begin{aligned} \begin{aligned} \textsf{V}(\textsf{S},\textsf{E},\textsf{I},\textsf{R})&=\bigg (- \ln \textsf{S}- a_1\ln \textsf{E}-a_2 \ln \textsf{I}-a_3\ln \textsf{R}+\zeta \int _{0}^{t}\textsf{I}(s)ds\bigg )\\ {}&-\ln \textsf{S}+\zeta \int _{t}^{t}\textsf{I}(s)ds-\ln \textsf{R}-\ln \textsf{E}+\frac{1}{1+\rho }(\textsf{E}+\textsf{R}+\textsf{S}+\textsf{I})^{1+\rho }\\&=\sum _{i=1}^5\textsf{V}_5, \end{aligned} \end{aligned}$$

where It is prominent to mention that $\textsf{V}$ is a function of the state variables which is defined for all possible values of the state variables. Further, this function has the property that it approaches $+\infty$ as the state variables go to their limits and $||(\textsf{S}, \textsf{E}, \textsf{I}, \textsf{R})|| \rightarrow \infty$.

Let us consider the initial data $(\textsf{S}_0, \textsf{E}_0, \textsf{I}_0, \textsf{R}_0)$ from the space ${\mathbb {R}}_+^4$, and subject this data, the function $\widetilde{V}$ (being a function of the state variables) will take the form:

28 $$\begin{aligned} \begin{aligned} \widetilde{V}&=\bigg (-\ln \textsf{S}-a_1\ln \textsf{R}- a_2\ln \textsf{E}-a_3 \ln \textsf{I}+\zeta \int _{0}^{t}\textsf{I}(s)ds\bigg )-\ln \textsf{S}\\ {}&-\ln \textsf{S}-\ln \textsf{R}+\zeta \int _{t}^{t}\textsf{I}(s)ds-\ln \textsf{E}+\frac{1}{1+\rho }(\textsf{E}+\textsf{S}+\textsf{R}+\textsf{I})^{1+\rho }\\&-\textsf{V}(\textsf{S}_0,\textsf{E}_0,\textsf{I}_0,\textsf{R}_0)\\&:=\sum _{i=1}^5\textsf{V}_i-\textsf{V}(\textsf{S}_0,\textsf{E}_0,\textsf{I}_0,\textsf{R}_0). \end{aligned} \end{aligned}$$

Here $(\textsf{S},\textsf{E},\textsf{I},\textsf{R})\in (\frac{1}{n},n)\times (\frac{1}{n},n)\times (\frac{1}{n},n)\times (\frac{1}{n},n)$ and $n>1$ is a so larger integer,

29 $$\begin{aligned} \begin{aligned} \textsf{V}_1&=- \ln \textsf{S}-a_1\ln \textsf{R}- a_2\ln \textsf{E}-a_3 \ln \textsf{I}+\zeta \int _{0}^{t}\textsf{I}(s)ds,\\ \textsf{V}_2&= \zeta \int _{t}^{t}\textsf{I}(s)ds-\ln \textsf{S},\\ \textsf{V}_3&=-\ln \textsf{E},\\ \textsf{V}_4&=-\ln \textsf{R},\\ \textsf{V}_5&=\frac{1}{1+\rho }(\textsf{E}+\textsf{S}+\textsf{R}+\textsf{I})^{1+\rho }, \end{aligned} \end{aligned}$$

where $\rho > 1$, and are subject to the condition(s) $\mu -\frac{\rho }{2}(\alpha _1^2\vee \alpha _2^2\vee \alpha _3^2\vee \alpha _4^2)>0,$ and $\delta =\frac{A\beta \epsilon }{\hat{\mu }\hat{\epsilon }\hat{\alpha }}-(\mu +\alpha +\gamma _3+\frac{v_4^2}{2})>0$. Thus

30 $$\begin{aligned} \begin{aligned} \textsf{A}=\sup \limits _{(\textsf{S},\textsf{E},\textsf{I},\textsf{R})\in {\mathbb {R}}_+^4}\bigg (-\frac{1}{4}\bigg [\mu -\frac{\rho }{2}(\alpha _1^2\vee \alpha _2^2\vee \alpha _3^2\vee \alpha _4^2)\bigg ]I^{\rho +1}\\ 2\mu +{{\textsf {N}}}+\varepsilon +a+B+\frac{\alpha _1^2}{2}+\frac{\alpha _2^2}{2}+\frac{\alpha _3^2}{2}\bigg ), \end{aligned} \end{aligned}$$

and

31 $$\begin{aligned} \begin{aligned} \textsf{B}&=\sup \limits _{(\textsf{S},\textsf{E},\textsf{I},\textsf{R})\in {\mathbb {R}}_+^4}\bigg \{A(\textsf{E}+\textsf{S}+\textsf{R}+\textsf{I})^\rho -\frac{1}{2}\bigg [\mu -\frac{\rho }{2}(\alpha _1^2\vee \alpha _2^2\vee \alpha _3^2\vee \alpha _4^2)\bigg ]\\&\times (\textsf{E}+\textsf{S}+\textsf{R}+\textsf{I})^{1+\rho }\bigg \}<\infty . \end{aligned} \end{aligned}$$

Using the well-known formula due to Itô to the function $\textsf{V}_1$, we obtained

32 $$\begin{aligned} \begin{aligned} L\textsf{V}_1&=-\frac{\mu {{\textsf {N}}}}{\textsf{S}}+\zeta \textsf{I}-\frac{\varepsilon \textsf{R}}{\textsf{S}}+(\mu +\omega ) +\frac{ \alpha _1^2}{2}-\frac{a_2\zeta \textsf{S}\textsf{I}}{\textsf{E}}+a_2(\mu +a)+\frac{a_2 \alpha _2^2}{2}\\ {}&-\frac{a_3a \textsf{E}}{\textsf{I}}+a_3(\gamma +\mu ) +\frac{a_3 \alpha _1^2}{2}-\frac{a_1\gamma \textsf{I}}{\textsf{R}}-\frac{a_1\omega \textsf{S}}{\textsf{R}}+a_1(\varepsilon +\mu )+\frac{a_1 \alpha _4^2}{2}+\zeta \textsf{I}-\zeta \textsf{I}(0)\,\\ {}&\le -3 \root 3 \of {\frac{\mu {{\textsf {N}}}}{\textsf{S}}\times \frac{a_2\textsf{S}\textsf{I}\times }{\textsf{E}}\textsf{I}\times \frac{a a_3\textsf{E}}{\textsf{I}}} +\bigg (\mu +\omega +\frac{\alpha _1^2}{2}\bigg )+a_2\bigg (\mu +a+\frac{\alpha _2^2}{2}\bigg )\\&+a_3\bigg (\gamma +\mu +\frac{\alpha _3^2}{2}\bigg )+a_1\bigg (\varepsilon +\mu +\frac{\alpha _4^2}{2}\bigg )-\frac{\varepsilon \textsf{R}}{\textsf{S}}-\frac{a_1\gamma \textsf{I}}{\textsf{R}}-\frac{a_1\omega \textsf{S}}{\textsf{R}}\\&\le -3 \root 3 \of {\mu {{\textsf {N}}}aa_2 a_3} +\mu +\bigg (\omega +\frac{\alpha _1^2}{2}\bigg )+a_2\bigg (\mu +a+\frac{\alpha _2^2}{2}\bigg )\\&+a_3\bigg (\gamma +\mu +\frac{\alpha _3^2}{2}\bigg )+a_1\bigg (\varepsilon +\mu +\frac{\alpha _4^2}{2}\bigg )-\frac{\varepsilon \textsf{R}}{\textsf{S}}-\frac{a_1\gamma \textsf{I}}{\textsf{R}}-\frac{a_1\omega \textsf{S}}{\textsf{R}}\\&= -3\mu \bigg ( \root 3 \of { \frac{{{\textsf {N}}}a}{\bigg (\mu +a+\frac{\alpha _2^2}{2}\bigg )\bigg (\gamma +\mu +\frac{\alpha _3^2}{2}\bigg )}} -1\bigg )+\bigg (\omega +\frac{\alpha _1^2}{2}\bigg )+a_1\bigg (\varepsilon +\mu +\frac{\alpha _4^2}{2}\bigg )\\&-\frac{\varepsilon \textsf{R}}{\textsf{S}}-\frac{a_1\gamma \textsf{I}}{\textsf{R}}-\frac{a_1\omega \textsf{S}}{\textsf{R}}\\&= -3\mu ( \root 3 \of {{\mathbb {R}}^s} -1)+\bigg (\omega +\frac{\alpha _1^2}{2}\bigg )+a_1\bigg (\varepsilon +\mu +\frac{\alpha _4^2}{2}\bigg )-\frac{\varepsilon \textsf{R}}{\textsf{S}}-\frac{a_1\gamma \textsf{I}}{\textsf{R}}-\frac{a_1\omega \textsf{S}}{\textsf{R}}. \end{aligned} \end{aligned}$$

Let

33 $$\begin{aligned} a_2\bigg (\mu +a+\frac{\alpha _2^2}{2}\bigg ) =a_3\bigg (\gamma +\mu +\frac{\alpha _3^2}{2}\bigg )=\mu . \end{aligned}$$

Similarly, we can get

34 $$\begin{aligned} \begin{aligned} L\textsf{V}_2=-\frac{\mu {{\textsf {N}}}}{\textsf{S}}+\zeta \textsf{I}-\frac{\varepsilon \textsf{R}}{\textsf{S}}+(\mu +\omega ) +\frac{\alpha _1^2}{2}-\zeta \textsf{I}(t)+\zeta \textsf{I}(0), \end{aligned} \end{aligned}$$

35 $$\begin{aligned} \begin{aligned} L\textsf{V}_3=-\frac{\zeta \textsf{S}\textsf{I}}{E}+(\mu +a)+\frac{\alpha _2^2}{2}, \end{aligned} \end{aligned}$$

36 $$\begin{aligned} L\textsf{V}_4=-\frac{\gamma \textsf{I}}{\textsf{R}}-\frac{\omega \textsf{S}}{\textsf{R}}+(\varepsilon +\mu )+\frac{ \alpha _4^2}{2}. \end{aligned}$$

37 $$\begin{aligned} \begin{aligned} L\textsf{V}_5&=(\textsf{E}+\textsf{S}+\textsf{R}+\textsf{I})^\rho [\mu {{\textsf {N}}}-\mu (\textsf{E}+\textsf{S}+\textsf{R}+\textsf{I}))\textsf{I}]+\frac{\rho }{2}(\textsf{E}+\textsf{S}+\textsf{R}+\textsf{I})^{-1+\rho }\\&\times (\alpha _1^2\textsf{S}^2\vee \alpha _2^2\textsf{E}^2 \vee \alpha _3^2\textsf{I}^2 \vee \alpha _4^2\textsf{R}^2),\\&\le (\textsf{E}+\textsf{S}+\textsf{R}+\textsf{I})^\rho [\mu {{\textsf {N}}}-\mu (\textsf{E}+\textsf{S}+\textsf{R}+\textsf{I})]+(\textsf{E}+\textsf{S}+\textsf{R}+\textsf{I})^{1+\rho }\frac{\rho }{2}(\alpha _1^2\vee \alpha _2^2 \vee \alpha _3^2\vee \alpha _4^2),\\&\le \mu {{\textsf {N}}}(\textsf{E}+\textsf{S}+\textsf{R}+\textsf{I})^\rho -(\textsf{E}+\textsf{S}+\textsf{R}+\textsf{I})^{1+\rho }\bigg [\mu -\frac{\rho }{2}(\alpha _1^2\vee \alpha _2^2 \vee \alpha _3^2\vee \alpha _4^2)\bigg ],\\&\le \textsf{B}-\frac{1}{2}\bigg [\mu -\frac{\rho }{2}(\alpha _1^2\vee \alpha _2^2 \vee \alpha _3^2\vee \alpha _4^2)\bigg ](\textsf{E}+\textsf{S}+\textsf{R}+\textsf{I})^{1+\rho },\\&\le -\frac{1}{2}\bigg [\mu -\frac{\rho }{2}(\alpha _1^2\vee \alpha _2^2 \vee \alpha _3^2\vee \alpha _4^2)\bigg ](\textsf{S}^{\rho +1}+\textsf{E}^{\rho +1}+\textsf{I}^{\rho +1}+\textsf{R}^{\rho +1})+\textsf{B}. \end{aligned} \end{aligned}$$

$\textsf{B}$ is given in Eq. (31).

From Eqs. (32)–(37), we follows

38 $$\begin{aligned} \begin{aligned} L\widetilde{\textsf{V}}&\le -3\mu ( \root 3 \of {{\mathbb {R}}^s} -1)+\bigg (\omega +\frac{\alpha _1^2}{2}\bigg )+a_1\bigg (\varepsilon +\mu +\frac{\alpha _4^2}{2}\bigg )-\frac{\varepsilon \textsf{R}}{\textsf{S}}-\frac{a_1\gamma \textsf{I}}{\textsf{R}}-\frac{a_1\omega \textsf{S}}{\textsf{R}}\\&-\frac{\mu {{\textsf {N}}}}{\textsf{S}}+\zeta \textsf{I}-\frac{\varepsilon \textsf{R}}{\textsf{S}}+(\mu +\omega ) +\frac{\alpha _1^2}{2}-\zeta \textsf{I}(t)+\zeta \textsf{I}(0)\\ {}&-\frac{\zeta \textsf{S}\textsf{I}}{E}+(\mu +a)+\frac{\alpha _2^2}{2}-\frac{\gamma \textsf{I}}{\textsf{R}}-\frac{\omega \textsf{S}}{\textsf{R}}+(\varepsilon +\mu )+\frac{\alpha _4^2}{2}\\ {}&+\textsf{B}-\frac{1}{2}\bigg [\mu -\frac{\rho }{2}(\alpha _1^2\vee \alpha _2^2 \vee \alpha _3^2\vee \alpha _4^2)\bigg ](\textsf{S}^{\rho +1}+\textsf{E}^{\rho +1}+\textsf{I}^{\rho +1}+\textsf{R}^{\rho +1}),\\&\le -3\mu ( \root 3 \of {{\mathbb {R}}^s} -1)+\bigg (\omega +\frac{\alpha _1^2}{2}\bigg )+a_1\bigg (\varepsilon +\mu +\frac{\alpha _4^2}{2}\bigg )-\frac{\varepsilon \textsf{R}}{\textsf{S}}-\frac{a_1\gamma \textsf{I}}{\textsf{R}}-\frac{a_1\omega \textsf{S}}{\textsf{R}}\\&-\frac{\mu {{\textsf {N}}}}{\textsf{S}}+\zeta \textsf{I}-\frac{\varepsilon \textsf{R}}{\textsf{S}}+\omega -\zeta \textsf{I}(t)+\zeta -\frac{\zeta \textsf{S}\textsf{I}}{E}\\ {}&+a-\frac{\gamma \textsf{I}}{\textsf{R}}-\frac{\omega \textsf{S}}{\textsf{R}} +\textsf{B}-\frac{1}{2}\bigg [\mu -\frac{\rho }{2}(\alpha _1^2\vee \alpha _2^2 \vee \alpha _3^2\vee \alpha _4^2)\bigg ](\textsf{S}^{\rho +1}+\textsf{E}^{\rho +1}+\textsf{I}^{\rho +1}+\textsf{R}^{\rho +1})\\&+\frac{\alpha _1^2}{2}+\frac{\alpha _2^2}{2}+\frac{\alpha _4^2}{2}. \end{aligned} \end{aligned}$$

Let $0<\xi$, and consider a closed bounded set in the form of

$$\begin{aligned} \textsf{D}=\Bigg \{(\textsf{S},\textsf{E},\textsf{I},\textsf{R})\in {\mathbb {R}}_+^4:\frac{1}{\xi }\ge \textsf{S}\ge \xi , \frac{1}{\xi }\ge \textsf{E}\ge \xi , \xi ^2 \le I\le \frac{1}{\xi ^2}, \xi ^3 \le \textsf{R}\le \frac{1}{\xi ^3}\Bigg \}. \end{aligned}$$

Over the complement of this set, that is ${\mathbb {R}}_+^4\backslash D$, we have the following inequalities:

39 $$\begin{aligned} -\frac{\mu {{\textsf {N}}}}{\xi }+\textsf{G}\le -1, \end{aligned}$$

40 $$\begin{aligned} -\mu {{\textsf {N}}}+\textsf{G}\le -1, \end{aligned}$$

41 $$\begin{aligned} -\mu {{\textsf {N}}}+\xi (1+c_3) + \textsf{A}\le -1, \end{aligned}$$

42 $$\begin{aligned} -\frac{\gamma }{\xi }+\textsf{G}\le -1, \end{aligned}$$

43 $$\begin{aligned} -\frac{\varepsilon }{\xi }+\textsf{G}\le -1, \end{aligned}$$

44 $$\begin{aligned} -\frac{1}{4}\bigg [\mu -\frac{\rho }{2}(\alpha _1^2\vee \alpha _2^2 \vee \alpha _3^2\vee \alpha _4^2)\bigg ]\frac{1}{\xi ^{\rho +1}}+\textsf{G}\le -1, \end{aligned}$$

45 $$\begin{aligned} -\frac{1}{4}\bigg [\mu -\frac{\rho }{2}(\alpha _1^2\vee \alpha _2^2 \vee \alpha _3^2\vee \alpha _4^2)\bigg ]\frac{1}{\xi ^{2(1+\rho )}}+\textsf{G}\le -1, \end{aligned}$$

46 $$\begin{aligned} -\frac{1}{4}\bigg [\mu -\frac{\rho }{2}(\alpha _1^2\vee \alpha _2^2 \vee \alpha _3^2\vee \alpha _4^2)\bigg ]\frac{1}{\xi ^{3(1+\rho )}}+\textsf{G}\le -1. \end{aligned}$$

Where

47 $$\begin{aligned} \textsf{G}= \sup \limits _{(\textsf{S},\textsf{E},\textsf{I},\textsf{R})\in {\mathbb {R}}_+^4}\bigg \{ c_1 \zeta I-\frac{1}{4}\bigg [\mu -\frac{\rho }{2}(\alpha _1^2\vee \alpha _2^2 \vee \alpha _3^2\vee \alpha _4^2)\bigg ]I^{\rho +1}\bigg ]\nonumber \\ +3\mu +\omega +a+\varepsilon +\textsf{B}+\frac{\alpha _2^2}{2} +\frac{\alpha _1^2}{2}+\frac{\alpha _4^2}{2}\bigg \}. \end{aligned}$$

We need to show that $L\widetilde{V}\le -1$ for any solution ${\mathcal {Q}}=(\textsf{S}, \textsf{E}, \textsf{I}, \textsf{R})\in {\mathbb {R}}_+^4 \backslash \textsf{D},$ and ${\mathbb {R}}_+^4 \backslash \textsf{D}=[\bigcup _{i=1}^8 \textsf{D}_{i}],$ where

48 $$\begin{aligned} \begin{aligned}{}&\textsf{D}_1=\bigg \{(\textsf{S},\textsf{E},\textsf{I},\textsf{R})\in {\mathbb {R}}_+^4; 0<\textsf{S}<\xi \bigg \},\\&\textsf{D}_2=\bigg \{(\textsf{S},\textsf{E},\textsf{I},\textsf{R}) \in {\mathbb {R}}_+^4; 0<\textsf{E}<\xi \bigg \},\\ {}&\textsf{D}_3=\bigg \{(\textsf{S},\textsf{E},\textsf{I},\textsf{R}) \in {\mathbb {R}}_+^4; 0<\textsf{I}<\xi ^2, \textsf{E}\ge \xi \bigg \},\\ {}&\textsf{D}_4=\bigg \{(\textsf{S},\textsf{E},\textsf{I},\textsf{R}) \in {\mathbb {R}}_+^4; 0<\textsf{R}<\xi ^3, \textsf{I}\ge \xi ^2 \bigg \},\\&\textsf{D}_5=\bigg \{(\textsf{S},\textsf{E},\textsf{I},\textsf{R}) \in {\mathbb {R}}_+^4; \textsf{S}>\frac{1}{\xi }\bigg \},\\ {}&\textsf{D}_6=\bigg \{(\textsf{S},\textsf{E},\textsf{I},\textsf{R}) \in {\mathbb {R}}_+^4; \textsf{E}>\frac{1}{\xi }\bigg \},\\&\textsf{D}_7=\bigg \{(\textsf{S},\textsf{E},\textsf{I},\textsf{R}) \in {\mathbb {R}}_+^4; \textsf{I}>\frac{1}{\xi ^2}\bigg \},\\ {}&\textsf{D}_8=\bigg \{(\textsf{S},\textsf{E},\textsf{I},\textsf{R}) \in {\mathbb {R}}_+^4; \textsf{R}>\frac{1}{\xi ^3}\bigg \}. \end{aligned} \end{aligned}$$

**Case 1.** If $(\textsf{S},\textsf{E},\textsf{I},\textsf{R})\in \textsf{D}_1$, then by Eq. (38), we get

49 $$\begin{aligned} \begin{aligned} L\widetilde{V}&\le -3\mu ( \root 3 \of {{\mathbb {R}}^s} -1)+\bigg (\omega +\frac{\alpha _1^2}{2}\bigg )+a_1\bigg (\varepsilon +\mu +\frac{\alpha _4^2}{2}\bigg )-\frac{\varepsilon \textsf{R}}{\textsf{S}}-\frac{a_1\gamma \textsf{I}}{\textsf{R}}-\frac{a_1\omega \textsf{S}}{\textsf{R}}\\&-\frac{\mu {{\textsf {N}}}}{\textsf{S}}+\zeta \textsf{I}-\frac{\varepsilon \textsf{R}}{\textsf{S}}+\omega -\zeta \textsf{I}(t)+\zeta -\frac{\zeta \textsf{S}\textsf{I}}{E}\\ {}&+a-\frac{\gamma \textsf{I}}{\textsf{R}}-\frac{\omega \textsf{S}}{\textsf{R}} +\textsf{B}-\frac{1}{2}\bigg [\mu -\frac{\rho }{2}(\alpha _1^2\vee \alpha _2^2 \vee \alpha _3^2\vee \alpha _4^2)\bigg ](\textsf{S}^{\rho +1}+\textsf{E}^{\rho +1}+\textsf{I}^{\rho +1}+\textsf{R}^{\rho +1})\\&+\frac{\alpha _1^2}{2}+\frac{\alpha _2^2}{2}+\frac{\alpha _4^2}{2},\\&\le -3\mu ( \root 3 \of {{\mathbb {R}}^s} -1)+\bigg (\omega +\frac{\alpha _1^2}{2}\bigg )+a_1\bigg (\varepsilon +\mu +\frac{\alpha _4^2}{2}\bigg )-\frac{\varepsilon \textsf{R}}{\textsf{S}}-\frac{a_1\gamma \textsf{I}}{\textsf{R}}-\frac{a_1\omega \textsf{S}}{\textsf{R}}\\&+\zeta \textsf{I}-\frac{\varepsilon \textsf{R}}{\textsf{S}}+\omega -\zeta \textsf{I}(t)+\zeta -\frac{\zeta \textsf{S}\textsf{I}}{E}\\&+a-\frac{\gamma \textsf{I}}{\textsf{R}}-\frac{\omega \textsf{S}}{\textsf{R}} +\textsf{B}-\frac{1}{2}\bigg [\mu -\frac{\rho }{2}(\alpha _1^2\vee \alpha _2^2 \vee \alpha _3^2\vee \alpha _4^2)\bigg ](\textsf{S}^{\rho +1}+\textsf{E}^{\rho +1}+\textsf{I}^{\rho +1}+\textsf{R}^{\rho +1})\\&+\frac{\alpha _1^2}{2}+\frac{\alpha _2^2}{2}+\frac{\alpha _4^2}{2}-\frac{\mu {{\textsf {N}}}}{\xi }. \end{aligned} \end{aligned}$$

If we use inequality (39), we can reach to the conclusion that $-1\ge L\widetilde{V}$ for all ${\mathcal {Q}}\in \textsf{D}_1$.

Further, with the help of inequalities (40), (41) and (42), and following a similar approach as that of **Case 1**, one can easily prove that $-1\ge \widetilde{V}$ for all ${\mathcal {Q}}\in \textsf{D}_2, \textsf{D}_3$ and $\textsf{D}_4$.

**Case 2**: If ${\mathcal {Q}}\in \textsf{D}_5$, then by Eq.(38), we get

50 $$\begin{aligned} \begin{aligned} L\widetilde{V}&\le -3\mu ( \root 3 \of {{\mathbb {R}}^s} -1)+\bigg (\omega +\frac{\alpha _1^2}{2}\bigg )+a_1\bigg (\varepsilon +\mu +\frac{\alpha _4^2}{2}\bigg )-\frac{\varepsilon \textsf{R}}{\textsf{S}}-\frac{a_1\gamma \textsf{I}}{\textsf{R}}-\frac{a_1\omega \textsf{S}}{\textsf{R}}\\&-\frac{\mu {{\textsf {N}}}}{\textsf{S}}+\zeta \textsf{I}-\frac{\varepsilon \textsf{R}}{\textsf{S}}+\omega -\zeta \textsf{I}(t)+\zeta -\frac{\zeta \textsf{S}\textsf{I}}{E}\\ {}&+a-\frac{\gamma \textsf{I}}{\textsf{R}}-\frac{\omega \textsf{S}}{\textsf{R}} +\textsf{B}-\frac{1}{2}\bigg [\mu -\frac{\rho }{2}(\alpha _1^2\vee \alpha _2^2 \vee \alpha _3^2\vee \alpha _4^2)\bigg ](\textsf{S}^{\rho +1}+\textsf{E}^{\rho +1}+\textsf{I}^{\rho +1}+\textsf{R}^{\rho +1})\\&+\frac{\alpha _1^2}{2}+\frac{\alpha _2^2}{2}+\frac{\alpha _4^2}{2},\\&\le -3\mu ( \root 3 \of {{\mathbb {R}}^s} -1)+\bigg (\omega +\frac{\alpha _1^2}{2}\bigg )+a_1\bigg (\varepsilon +\mu +\frac{\alpha _4^2}{2}\bigg )-\frac{\varepsilon \textsf{R}}{\textsf{S}}-\frac{a_1\gamma \textsf{I}}{\textsf{R}}-\frac{a_1\omega \textsf{S}}{\textsf{R}}\\&-\frac{\mu {{\textsf {N}}}}{\textsf{S}}+\zeta \textsf{I}-\frac{\varepsilon \textsf{R}}{\textsf{S}}+\omega -\zeta \textsf{I}(t)+\zeta -\frac{\zeta \textsf{S}\textsf{I}}{E}\\ {}&+a-\frac{\gamma \textsf{I}}{\textsf{R}}+\textsf{B}-\frac{1}{2}\bigg [\mu -\frac{\rho }{2}(\alpha _1^2\vee \alpha _2^2 \vee \alpha _3^2\vee \alpha _4^2)\bigg ](\textsf{S}^{\rho +1}+\textsf{E}^{\rho +1}+\textsf{I}^{\rho +1}+\textsf{R}^{\rho +1})\\&+\frac{\alpha _1^2}{2}+\frac{\alpha _2^2}{2}+\frac{\alpha _4^2}{2}. \end{aligned} \end{aligned}$$

Here again, if we use inequality (43), we can reach to the conclusion that $-1\ge L\widetilde{V}$ for all ${\mathcal {Q}}\in \textsf{D}_5$.

Moreover, with the help of inequalities (44), (45) and (46), and following a similar approach as that of **Case 2**, one can easily prove that $-1\ge \widetilde{V}$ for all ${\mathcal {Q}}\in \textsf{D}_6, \textsf{D}_7$ and $\textsf{D}_8$.

In the conclusion, we have

$$\begin{aligned} L\widetilde{V}< -\textbf{W} < 0~\hbox {for all}~{\mathcal {Q}} \in \textbf{R}_{+}^{4} \backslash \textsf{D}, \end{aligned}$$

which are sufficient in proving the condition (2) in Lemma 3 and thus, system (8) has the property of ergodicity and hence the theorem. $\square$

## Numerical simulations

In the previous section, we obtained some important theoretical results based on the dynamic perspectives of the deterministic and stochastic models. In this part, we intend to verify these results through simulations. We will employ the standard higher ordered Milstein’s method^33^ to discretized system (8), Milstein’s method is an extension of the Euler-Maruyama method, aiming to improve the accuracy of numerical solutions for SDEs. The associated discretization equations are given by:

51 $$\begin{aligned} \begin{aligned} \textsf{S}_{i+1}&=\textsf{S}_{i}+\bigg [\mu {{\textsf {N}}}-\zeta \textsf{S}_{i} \textsf{I}_{i}+\varepsilon \textsf{R}_{i}-(\mu +\omega ) \textsf{S}_{i}\bigg ]\bigtriangleup t+\alpha _{1} \textsf{S}_{i} \sqrt{\bigtriangleup t}\xi _{1,i}+\frac{\alpha _{1}^2}{2}\textsf{S}_{i}(\xi _{1,i}^2-1)\bigtriangleup t,\\ \textsf{E}_{i+1}&=\textsf{E}_{i}+\bigg [\zeta \textsf{S}_{i} \textsf{I}_{i}-(\mu +a)\textsf{E}_{i}\bigg ]\bigtriangleup t+\alpha _{2} \textsf{E}_{i}\sqrt{\bigtriangleup t}\xi _{1,i}+\frac{\alpha _{2}^2}{2}\textsf{E}_{i}(\xi _{1,i}^2-1)\bigtriangleup t,\\ \textsf{I}_{i+1}&=\textsf{I}_{i}+\bigg [a \textsf{E}_{i}-(\gamma +\mu ) \textsf{I}_{i}\bigg ]\bigtriangleup t+\alpha _{3} I_{i}\sqrt{\bigtriangleup t}\xi _{1,i}+\frac{\alpha _{3}^2}{2}I_{i}(\xi _{1,i}^2-1)\bigtriangleup t,\\ \textsf{R}_{i+1}&=\textsf{R}_{i}+\bigg [\gamma \textsf{I}_{i}+\omega \textsf{S}_{i}-(\varepsilon +\mu ) \textsf{R}_{i}\bigg ]\bigtriangleup t+\alpha _{4} \textsf{R}_{i}\sqrt{\bigtriangleup t}\xi _{1,i}+\frac{\alpha _{4}^2}{2}\textsf{R}_{i}(\xi _{1,i}^2-1)\bigtriangleup t. \end{aligned} \end{aligned}$$

where the term $\Delta t$ represents a positive uniform time-step and $\xi _i,$ such that $i=1,\cdots ,4$ are the independent Gaussian random variables that follow the Gaussian distribution ${{\textsf {N}}}(0,1)$ for $i=1,2, 3, 4$. The set for the time is tested [0, 5000], and $\Delta =0.5$.

### Simulations based on sensing radius of nodes

**Figure 3:**
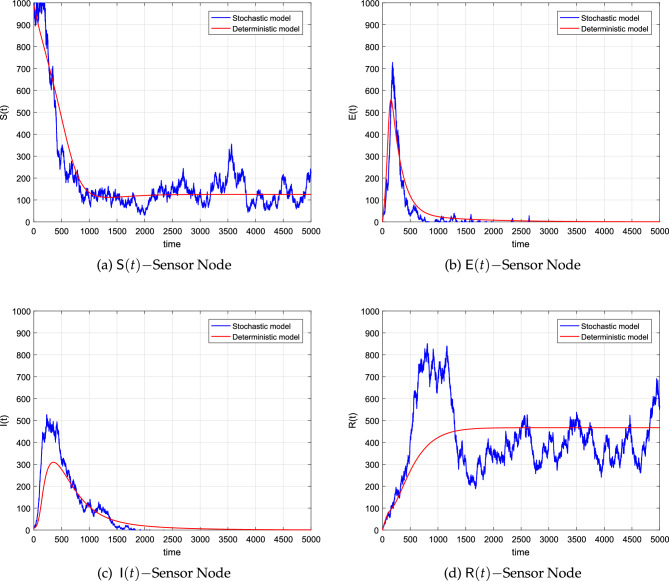
The corresponding simulations of the system (8) and the deterministic system (1).

We have seen in the analysis part of the study that the condition ${\mathbb {R}}_s$ acts as a threshold parameter for the model, that is, the dynamics of the solution to the model can be completely specified in terms of that parameter. Under the conditions of ${\mathbb {R}}_s<1$, the infected nodes will approach 0 in the long run. The integral curves of the deterministic system will reach WFE and the curves of the stochastic model will oscillate around the stated equilibrium point. Simulations further confirm that the virus will eventually leave the WSN when we keep ${\mathbb {R}}_s<1$ and analytically, it is proved in Theorem 4. Graphically, these conclusions could be confirmed from Fig. 3a–d which are the results of the simulations. The values of the parameters as well as the initial size of the nodes are shown in Table 2. The simulation was carried out by implementing the scheme (obtained by following the method presented in the previous section) within MATLAB (R2017a).

Figure 3 shows that by using these values of the parameters, no matter what the value of $\textsf{S}_0$, the susceptible nodes will reach the component $\textsf{S}^0$ of susceptibles in the WFE. Similarly, in the initial course of worm spread, the size of the exposed and virus-infected nodes will tend to grow. After achieving the respective maximum values, the size of these nodes will exhibit a decline and finally reach zero as $t\rightarrow \infty$. This behavior could be easily noticed from Fig. 3b,c where we plotted sample solution curves of the virus-infected compartment utilizing both the stochastic and deterministic models. Finally 3d reflects the behavior of the recovered nodes which initially showed an increase in the size and finally reached the respective fixed point.

The figures further elaborate that under the condition of ${\mathbb {R}}_s< 1$, the curves reaching non-trivial components of the WFE fluctuate more compared to those approaching the trivial components. The figures also suggest that the method converges fast to the desired equilibrium for any discretization.

**Table 2:** Values of the parametric for simulating models (1) and (8) to explain the behavior of extinction of the worms within the networks.

Parameter	Value	Source
${{\textsf {N}}}$	$1 \times 10^{3}$	^29^
*a*	$4\times 10^{-3}$	^29^
$\mu$	$1\times 10^{-3}$	^29^
$\beta$	$7\times 10^{-4}$	Assumed
*L*	10	^29^
$\omega$	$1\times 10^{-3}$	^29^
$\gamma$	$2\times 10^{-3}$	^29^
$\varepsilon$	$3\times 10^{-4}$	^29^
$\textsf{S}(0)$	990	^29^
$\textsf{E}(0)$	1.00	^29^
$\textsf{I}(0)$	9.00	^29^
$\textsf{R}(0)$	0.00	^29^
$\alpha _1$	0.50	Assumed
$\alpha _2$	0.30	Assumed
$\alpha _3$	0.45	Assumed
$\alpha _4$	0.40	Assumed

### Simulations regarding nodes distribution

We stated and proved Theorem 5 which ensures the existence of a worm within the network at any time *t*. To simulate both the deterministic and stochastic model for this scenario, we shall assume values of the parameters as well as the initial value from Table 3. By using these values of the parameters, we calculated ${\mathbb {R}}^s$ which was noticed greater than one. When we simulated the models several times, we observed that the curves of the deterministic system approach the worm-present equilibrium. Likewise, the stochastic curves approach the EE but not actually as in the case of deterministic. Such curves will fluctuate in the vicinity of the deterministic curves. Taking into consideration both the temporal and spatial values of the variables and parameters, the dynamics of the curves suggest the presence of the worm in the networks, and hence a control strategy should be followed to reduce the spread.

**Figure 4:**
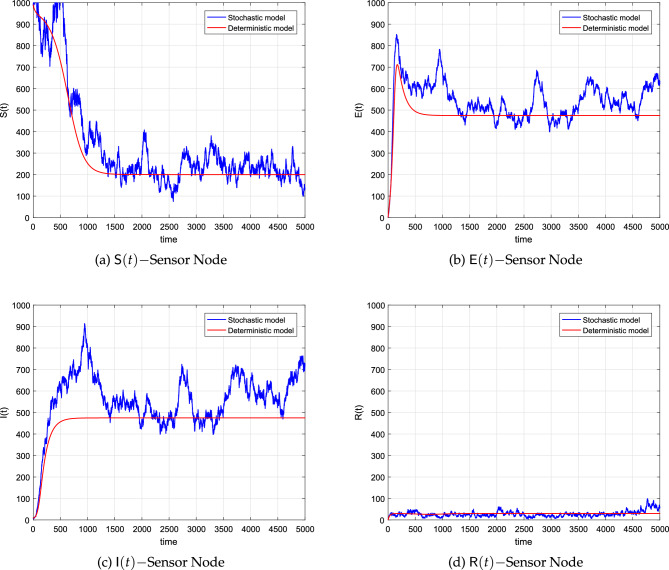
The corresponding simulations of the system (8) and the deterministic system (1).

Figure 4 show a drastic decrease in the size of the susceptible nodes and then reach a steady state as time evolves. Figure 4b,c show the dynamics of the exposed and infected nodes under the condition of ${\mathbb {R}}^s>1$. The plots indicate that the worms initially spread almost at an exponential rate and finally approached the respective fixed points. Finally, we presented the dynamics of the removed nodes in Fig. 4d where the curves almost show a constant behavior. All of these figures show that the worms will remain in the network, resulting in a stable EE. Furthermore, it has been discovered that increasing the connectedness of sensor nodes leads to enhanced network connectivity.

Here again, the convergence of the curves is independent of the discretization and this shows the beauty of the proposed scheme. All of the deterministic curves reach the desired equilibria and the associated stochastic curves fluctuate around the deterministic curves. Due to the high intensity of the noises, the amplitude of the oscillation of the stochastic curves is observed large compared to curves with small values of the noises.

**Table 3:** The parametric values of models (1), and (8).

Parameter	Value	Source
${{\textsf {N}}}$	1000	^29^
*a*	0.004	^29^
$\mu$	0.020	Assumed
$\beta$	0.0007	Assumed
*L*	10.00	^29^
$\omega$	0.001	^29^
$\gamma$	0.002	^29^
$\varepsilon$	0.0003	^29^
$\textsf{S}(0)$	990.0	^29^
$\textsf{E}(0)$	1.000	^29^
$\textsf{I}(0)$	9.000	^29^
$\textsf{R}(0)$	0.000	^29^
$\alpha _1$	0.300	Assumed
$\alpha _2$	0.200	Assumed
$\alpha _3$	0.400	Assumed
$\alpha _4$	0.450	Assumed

#### Histogram of the node distribution

**Figure 5:**
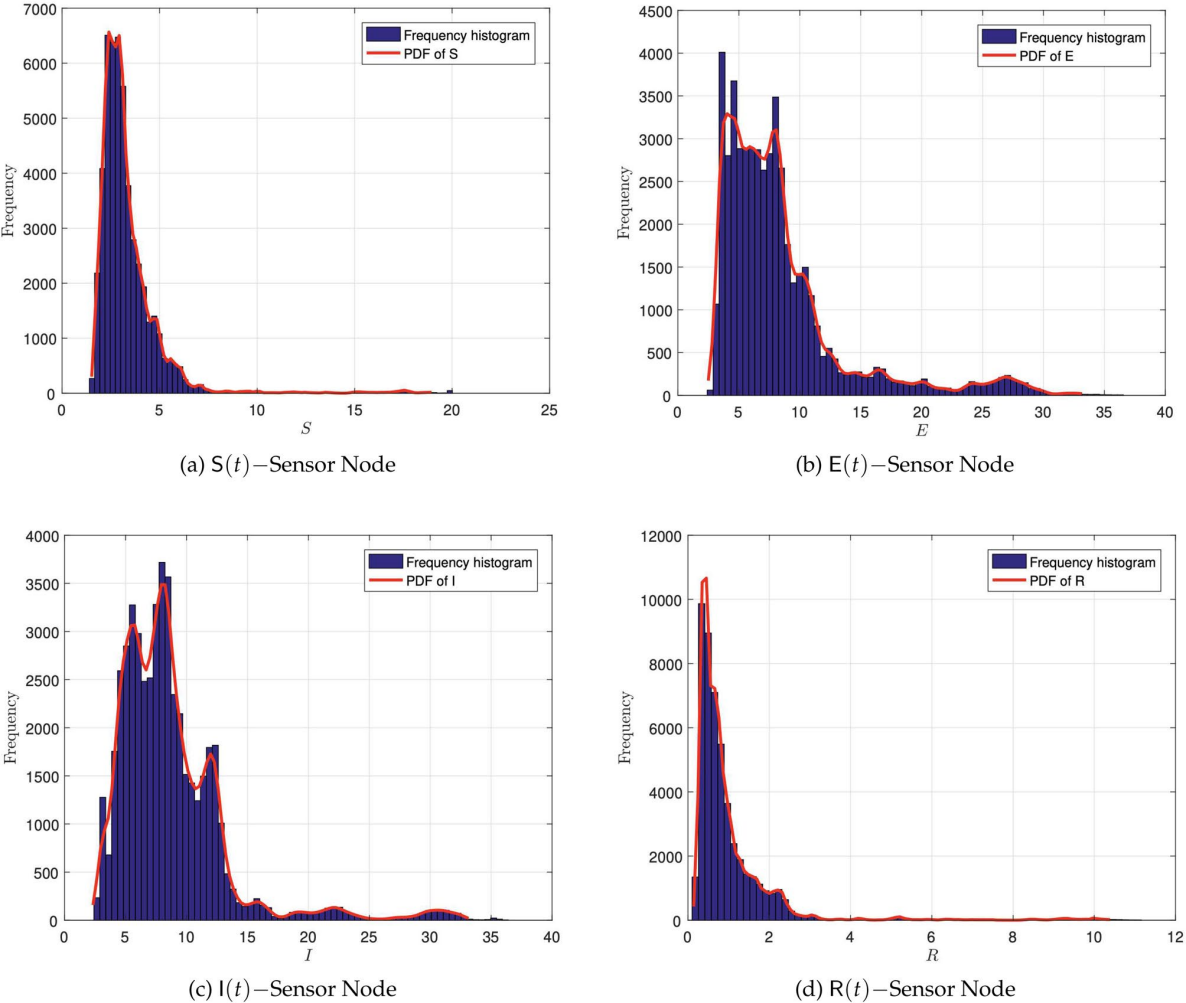
The histogram of the solution and the corresponding marginal density function curve for each sensor node.

Next, to specifically describe the effects of each noise term on the dynamic behavior of the system (8), we assume that the system is influenced by only one random noise. Figure 5a–d illustrate the impact of noise strength on the fluctuations of each population. It can be observed that low-strength noise results in minimal population fluctuations, while high-strength noise causes significant oscillations and maintains populations at certain levels. Additionally, the histograms of the solutions and the corresponding marginal density function curves for each population are presented. The associated values of the parameters and initial conditions of the state variables about the model (8) are provided in Table 4.

**Table 4:** Values of the parametric for simulating systems (1), and (8) to explain the behavior of the persistence of the worms within the networks.

Parameter	Value	Source
${{\textsf {N}}}$	$1\times 10^{2}$	Assumed
*a*	$1\times 10^{-2}$	Assumed
$\mu$	$2\times 10^{-2}$	Assumed
$\beta$	$7\times 10^{-4}$	Assumed
*L*	1	Assumed
$\omega$	$2\times 10^{-3}$	Assumed
$\gamma$	$2\times 10^{-3}$	Assumed
$\varepsilon$	$1\times 10^{-4}$	Assumed
$\textsf{S}(0)$	990	^29^
$\textsf{E}(0)$	1	^29^
$\textsf{I}(0)$	9	^29^
$\textsf{R}(0)$	0	^29^
$\alpha _1$	0.3	Assumed
$\alpha _2$	0.2	Assumed
$\alpha _3$	0.4	Assumed
$\alpha _4$	0.45	Assumed

### The impact of noise on system (8)

**Figure 6:**
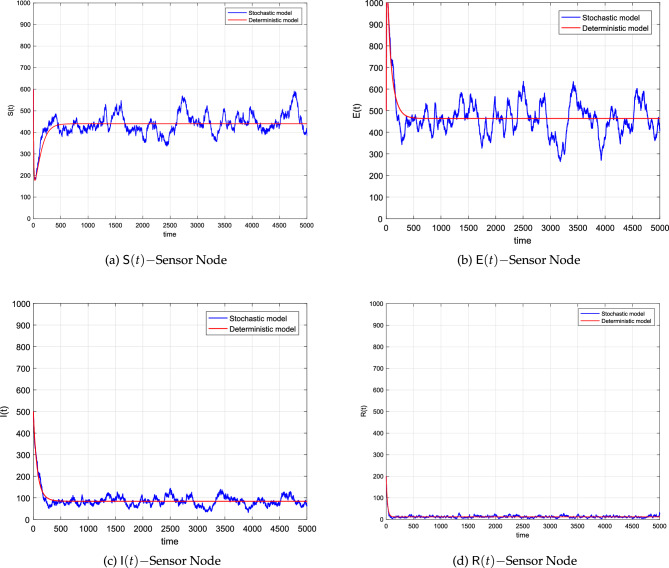
The affects of noise intensities on $\textsf{S}(t)$, $\textsf{E}(t)$, $\textsf{I}(t)$, and $\textsf{R}(t)$.

This section provides the effect of intensities on the dynamics of the worm-infected nodes in connection with system (8). The impact of noise intensities was plotted in Fig. 6a–d where it can be deduced that if one increases $\alpha _i$, resultantly the worm-infected nodes will tend to extinct out of the network. We conducted observations on the impact of the radius of nodes on the dynamic behavior of the model. It was noticed that the optimization of the threshold value (flexibility shifts) has a significant influence on various aspects, including the enhancement of the lifetime of networks, elimination of the malware from the networks, and controlling the spread of the malware. The remaining parameters and initial values of the system (8) were obtained from Table 3 and changed the initial values to $(\textsf{S},\textsf{E},\textsf{I},\textsf{R})=(600,500,500,200)$.

## Conclusion

In this study, we have examined a stochastic $\textsf{S}\textsf{E}\textsf{I}\textsf{R}\textsf{S}$ model to control worm spread in WSNs. The model incorporates stochastic components arising from environmental variability, which are represented as Gaussian white noise. We derived a set of sufficient conditions that determine the persistence or extinction of worm spread in WSNs in terms of the mean behavior. These conditions offer valuable insights into the long-term dynamics of the system and contribute to enhancing our understanding of worm propagation dynamics and control strategies in WSNs. At first, we mentioned that our model is globalized, positive, and feasible root by applying the the technique of Lyapunov function. After that, we have computed a threshold value of our stochastic system (8) and basic reproductive value ${\mathbb {R}}_0$ of the defined deterministic system (1) without oscillation of noises. We also concluded, if ${\mathbb {R}}_s<1$, the infection will die with sure probability, otherwise if ${\mathbb {R}}_0^s>1$, the infection lies in the mean of the WSNs. In the last, we have compared our obtained scheme results through simulations.

The extensive results obtained from this study provide evidence that the proposed model contributes to increased network lifetime and improved data efficiency in Wireless Sensor Networks. These findings have practical implications for software organizations, as they can utilize this knowledge to develop more effective antivirus software that can effectively restrict malware attacks in WSNs. Moreover, the investigation will assist end-users in recovering infected nodes and implementing antivirus software on sensor nodes with careful consideration, thereby strengthening the overall security framework to mitigate attacks.

Furthermore, future research directions can include the analysis of additional factors such as vaccinated and quarantined classes, as well as the inclusion of heterogeneous and mobile nodes. These considerations can enhance the model’s applicability and provide further insights into the dynamics of worm spread and mitigation strategies in WSNs.

## Data Availability

All data generated or analyzed during this study are included in this published article.
